# The Relationship Analysis between Water Injection and Microfacies of SHA1 Reservoir of Liao He Basin, China

**DOI:** 10.1155/2014/641571

**Published:** 2014-02-05

**Authors:** Qing Wang, Zhanguo Lu, Shiguang Guo, Chao Wang

**Affiliations:** ^1^Institute of Geology and Geophysics, Chinese Academy of Sciences, Beijing 100029, China; ^2^Beijing Orangelamp Geophysical Exploration Co., Ltd., Beijing 102200, China; ^3^ConocoPhillips School of Geology and Geophysics, University of Oklahoma, Norman, OK 73019, USA; ^4^China Oilfield Services Limited, Beijing 101149, China

## Abstract

SHA1 is the representative reservoir in Liao He Basin. Through the introduction of curvature displayed on the gray scale, we determine the substructure and fractures. Geostatistical inversion method is used to help study the porosity of reservoir. The relationship between interval transit times and resistivity among mudstone and sandstone, before and after water injection, is analyzed. The relationship between porosity and permeability and the relationship between porosity and impedance from core analysis were studied. Through the whole information above, we divide the microfacies of SHA1 reservoir to distributary channel, mouth bar, the leading edge thin sand, and prodelta mud. The water injections in different microfacies are studied. The distributary channel should be used by large distant injection wells or smaller injection pressure injection. The smaller distant injection wells or large injection pressure should be used in the mouth bar. The arrangement of well injection need consider the different sedimentary microfacies.

## 1. Introduction

**Figure 1:**
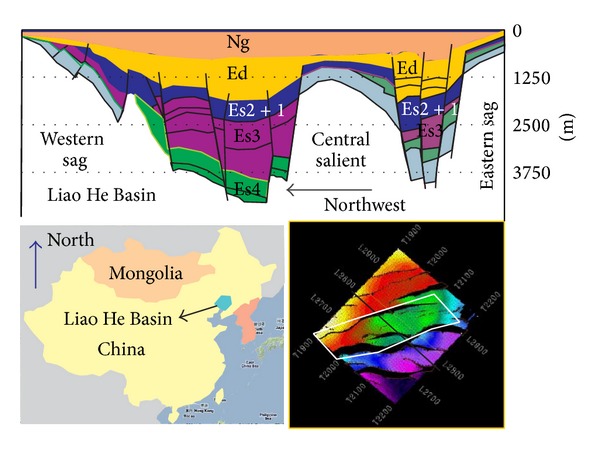
Location map showing the area of study. White circle is the study area; black line is seismic profile in Figure 2.

**Figure 2:**
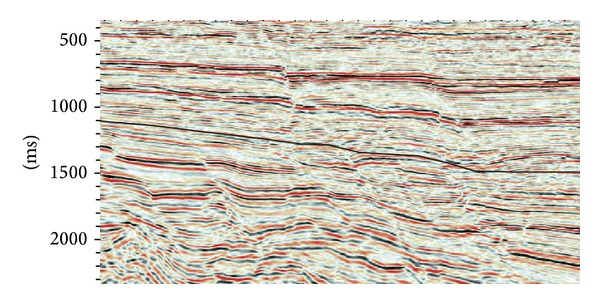
The target layer in black lines.

The reservoir position of SHA1 is located in southern section of the western slope of west Liao He sag basin. The overall structural form is sloping monoclinically from west to east [1, 2].  Figure 1 shows the location and geological layer in Es2+1. The white circle in the right side of Figure 1 indicates the study area, and the black line signs the seismic profile in Figure 2. In Figure 2, the black line shows the target layer we studied. SHA1 reservoir is a sandstone reservoir and shows high heterogeneity. Fault and fracture play pivotal roles on the accumulation and migration of oil and gas and the direction of water injection. This block develops the fan-delta front subfacies, with thick superimposing sand body and wide distribution area. However, because the strong reservoir heterogeneity, large differences in physical properties of the oil layers, complex oil-water relationship, and severe flooding, SHA1 region has some characteristics such as scattered distribution of residual oil, unclear understanding of the longitudinal residual oil, the uneven reserves producing, and with so many high-containing wells. It is hard to complete water-shutoff and separate injection [3]. So it results in low passing rate of stratified injection and the decreased utilization. Also the predecessors only divided this area into subfacies, not further exploring the relationship between sedimentary microfacies and injection. Water injection and development have been closely linked with the underground geological factors; therefore, the studies between microfacies and water injection are never stopped. It also becomes a major feature of the study [4]. The purpose is to guide the development of water well injection based on the knowledge of substructure and microfacies [5]. Substructure and microfacies are considered as the most typical characteristics which play a vital role in reservoir. The heterogeneity directly affects the direction and velocity of water injection.

The studies about this block are various and changed during twenty years. Yin Jixian et al. consider that this reservoir a rapid accumulation of fan delta sand body; the source came from both directions north and south. However, some scholars have different opinions; they believe that this block should be a braided river delta in SHA1 reservoir [6]. Regarding water injection, the regular movements of water injection in different microfacies are different because several of sedimentary environments, such as sand grain size, porosity, permeability, heterogeneity, connectivity, geometry, rhythm, structure, and tectonic features and other aspects. In order to study the relation between the water injection and microfacies, we used 3D curvature to detect substructure and field-scale fracture in SHA1 reservoir. Geostatistical inversion method is used to study the porosity destitution. The core and well analysis are used to help us fuilfull reservoir characteristic. Through analyzing the data from production well and water injection well, we analyze the relationship between water injection and microfacies. This workflow and methods should be used in the development of other high porosity and heterogeneity reservoirs in Liao He Basin, China.

## 2. Methods

**Figure 3:**
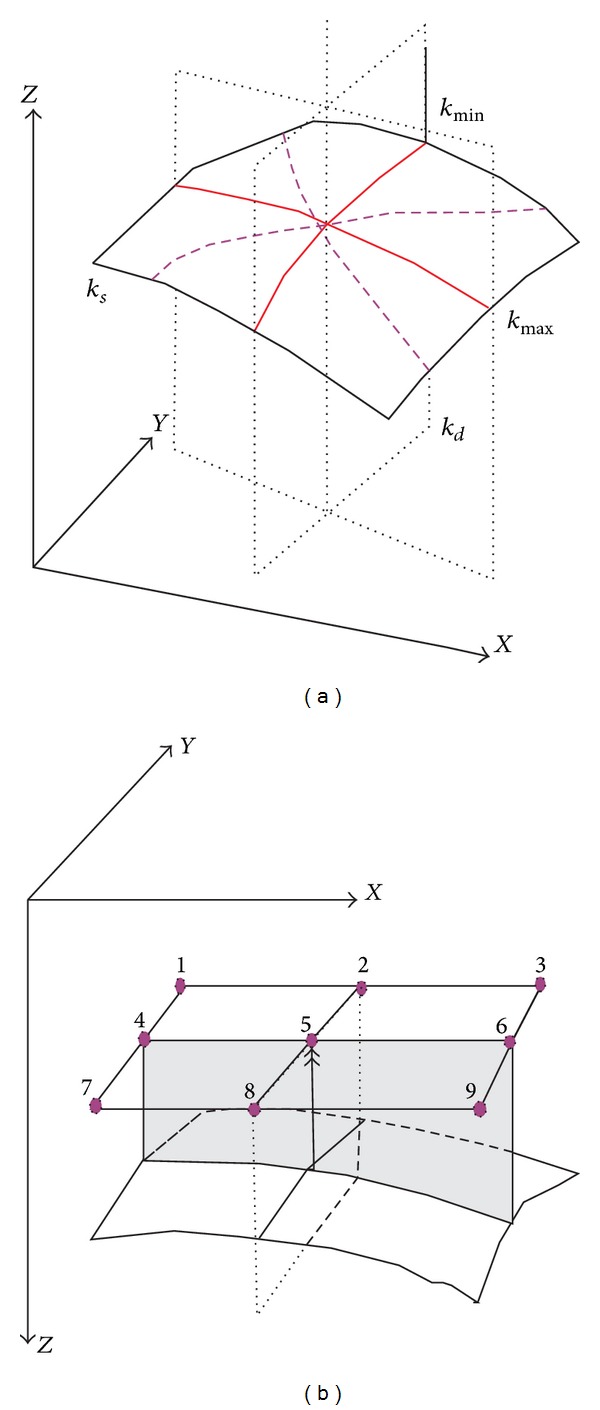
Sketch map and algorithm of 3D curvature [7].

Volume curvature is a description of how bent a curve is at a particular point on the curve. The smaller the radius of curvature, the more bent the curve and the larger the curvature. Curvature for subtle fault detection has the huge advantage over coherency result from its flat character. Because of its edge-detection capabilities, it can greatly improve the geophysicist's ability to rapidly map structural frame works and interpret details that are typically unrecognized in conventional amplitude data sets [7]. The volumetric curvature can overcome the vertical smearing and resolve excellent fine fault.  Figure 3(a) shows *X*, *Y*, and *Z* axes; *k*_min⁡_ denotes minimum curvature, *k*_max⁡_ denotes maximum curvature, *k*_*d*_ denotes dip curvature, and *k*_*s*_ denotes strike curvature.  Figure 3(b) shows 9 gridding nodes; they denote the gridding unit that was used in curvature calculation; node 5 is the location of curvature calculation. Furthermore, the less steeply dipping faults can be detected by volumetric curvature.

Geostatistical inversion method is a comprehensive utilization of seismic data and logging data; the inversion results not only satisfy the lateral continuity but also integrate the vertical high-resolution logging data. It provides a good tool for the reservoir prediction in the development stage [8]. The core of geostatistical inversion is random algorithm; the popular algorithms are sequential Gaussian simulation algorithm and Markov chain Monte Carlo algorithm. Among them, Markov chain Monte Carlo algorithm is later rise. Compared with the sequential Gaussian simulation algorithm, Markov chain has good features: the ability to unlimitedy approximating any complex distribution function, without waiting for the simulation parameters, obeys the Gauss distribution and after N iterations can always achieve the stationary distribution [9]. Therefore, this research uses geostatistical inversion to inverse porosity based on Markov chain Monte Carlo algorithm for reservoir prediction [10].

Core and log analysis support the correct information about mineral and responses in log curve [11, 12]. We analyzed the relationship between interval transit times and resistivity among mudstone and sandstone, before and after water injection. The relationship between porosity and permeability and the relationship between porosity and impedance from core analysis were studied. After the studies above, the geological reserves are calculated based on the formula as follows: *N* = 100∗*A*_0_∗*H*∗Φ∗*S*_0*i*_∗*ρ*_0_/*B*_0*i*_ (*N* is geological reserves, *A*_*o*_ is area of oil, *H* is the average effective thickness, Φ  is the average effective porosity, *S*_0*i*_ is average oil saturation, *ρ*_0_ is the average surface oil density, and *B*_0*i*_ is crude oil volume factor: 1.39). Moreover, based on the data from production well and water injection well, we analyze the relationship between water injection and microfacies.

## 3. Results

**Figure 4:**
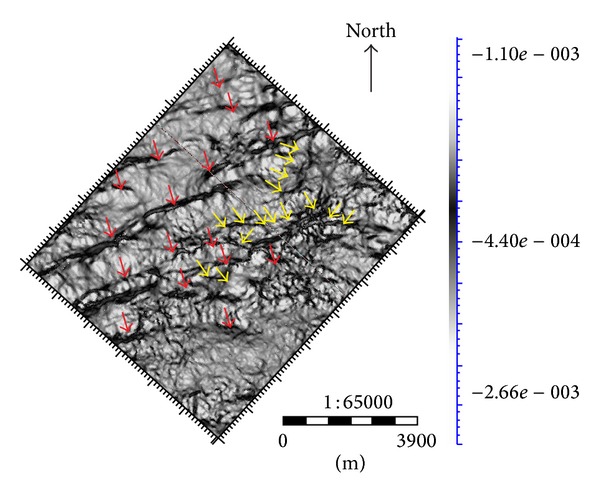
The substructure along the target layer in curvature attributes.

In order to detect more faint faults and fractures, the volumetric curvature attributes not only overcome the shortage of coherency but also make use of its advantages to find smaller scale faults and fracture near the main fault. In Figure 4, red arrows show us the main structure faults; yellow arrows indicate other fine faults and fractures which are hard to detect by coherence attributes. These tiny structures play an important role in the distribution and migration of oil.

**Figure 5:**
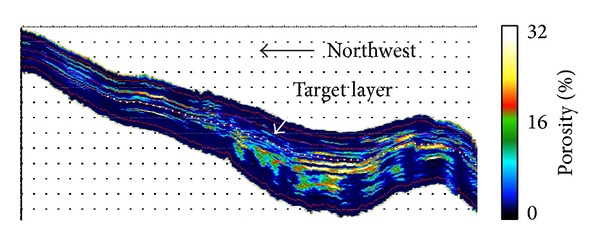
Porosity inversion profile.

**Figure 6:**
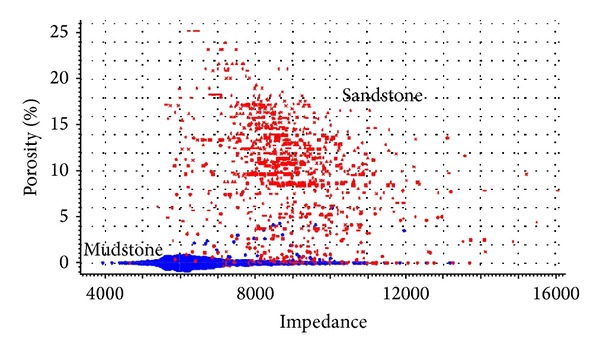
The relationship between porosity and p-impedance of sandstone and mudstone.

The porosity profile illuminated the distribution of porosity in Figure 5. The white note locates the target layer in SHA1 reservoir. We find that porosity in both water-bearing sandstone and in oil is high. According to the inversion data, the information about relationships of porosity and p-impendence between sandstone and mudstone was acquired in Figure 6. These achievements were used for reservoir and remaining oil characterization.

**Figure 7:**
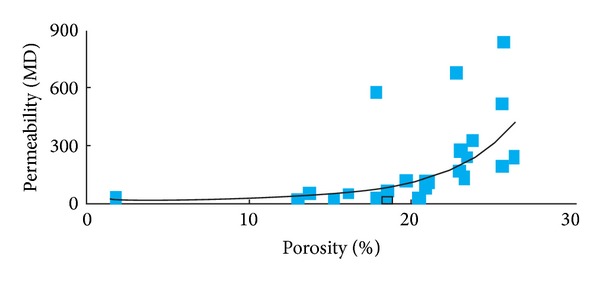
The relationship between porosity and permeability *K* = 0.707∗*e*(0.2412∗Φ).

**Figure 8:**
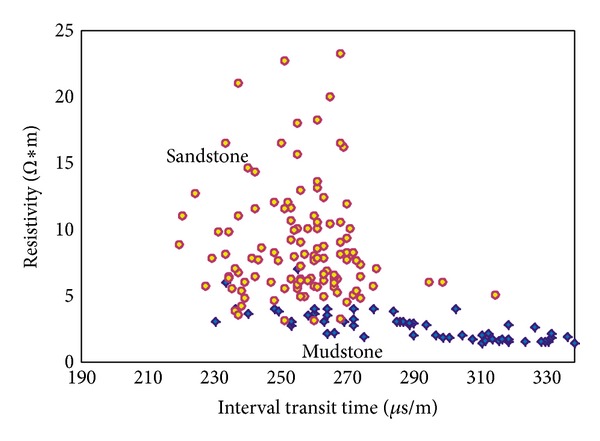
The relationship between interval transit times and resistivity.

Figure 7 shows the relationship between porosity and permeability. This information came from the laboratory core measurements and statistics. The relationship gives us the information about permeability of SHA1 reservoir.  Figure 8 indicates the relationship between interval transit times and resistivity among mudstone and sandstone; this information helps us to use resistivity log data to divide the lithofacies.

**Figure 9:**
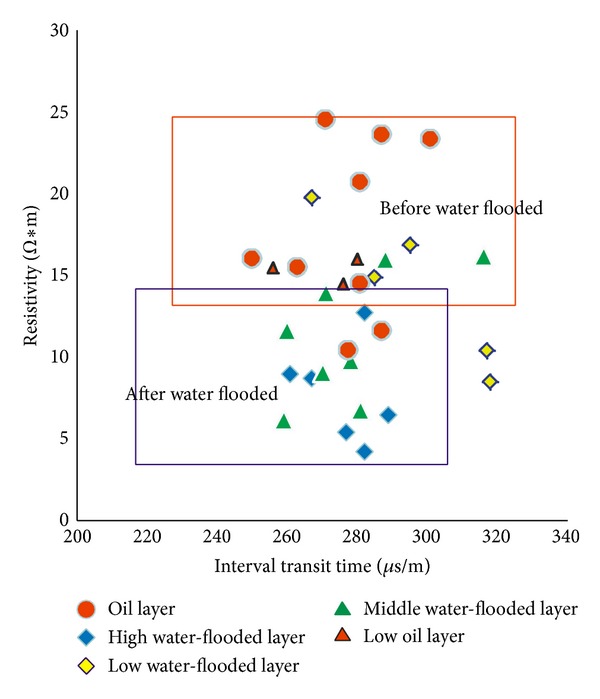
The relationship between interval transit times and resistivity before and after water injection.

In order to find the law of resistivity and interval transit times before and after water injection, we collect statistics the data from wells to solve this uncertainty.  Figure 9 shows us the details. High, middle, and low water-flooded layer and high, and low oil layer are illustrated. We find the resistivity becomes low after water injection (from 15–25 to 5–15 *Ω*∗m) and interval transit times changed little. According to the geological reserves formula, we calculate that the geological reserves are 3.05∗10^6^ *t*, the area of oil is 4.1 km^2^, and the average effective thickness is 8 meters.

**Figure 10:**
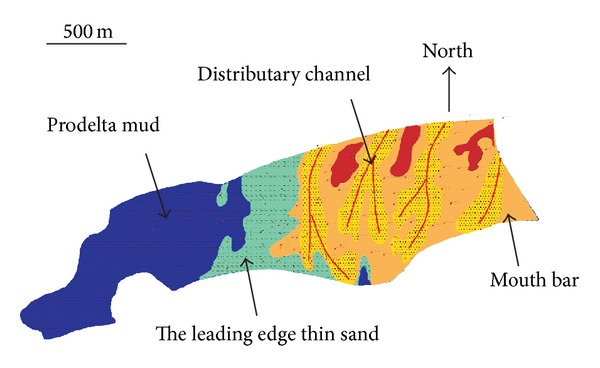
The microfacies of SHA1 reservoir.

**Figure 11:**
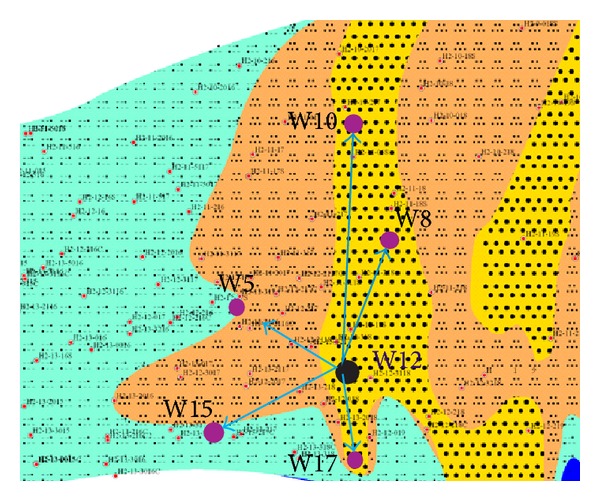
Distribution of injection wells and oil wells.

Through the whole information above, we divide the microfacies of SHA1 reservoir to distributary channel, mouth bar, the leading edge thin sand, and prodelta mud (Figure 10). In Figure 11, W12 is the injection wells; other wells are oil wells. The blue lines show the routes and direction of oil migration. The oil in distributary channel is firstly flooded and the velocity of migration is fast; however, in mouth, bar it is low.

## 4. Conclusion

The information of substructure is importantly useful to understand the reservoir. Curvature for subtle fault detection has the huge advantage to detect subtle fault and faint fracture. Geostatistical inversion method is suitable for use in the reservoir characteristics. They help to better predicate the oil and gas reservoir and analyze the possible distribution of residual oil. The porosity and permeability play a vital role in the distribution and migration of residual oil. However, the process of porosity, permeability, and lithofacies calculation should be combined with log and core data.

The relationship between water injection and sedimentary microfacies is close. Because of the good penetration and porosity, the water in distributary channel travels quickly along the river path but not uniform and the water in mouth bar or the leading edge thin sand travels slowly but uniform. Therefore, the water injection in distributary channel should be used from large distant injection wells or smaller injection pressure injection; the water injection in mouth bar and the leading edge thin sand should be use smaller distant injection wells or large water injection pressure. To the need for taking care of wells in the different sedimentary microfacies, the injection well should be arranged in a relatively uniform. The low penetration of microfacies, such as the estuarine sand bar and front sheet sand, helps to promote a uniform body of water.
